# Role of Dietary Protein and Thiamine Intakes on Cognitive Function in Healthy Older People: A Systematic Review

**DOI:** 10.3390/nu7042415

**Published:** 2015-04-02

**Authors:** Freda Koh, Karen Charlton, Karen Walton, Anne-Therese McMahon

**Affiliations:** School of Medicine, University of Wollongong, New South Wales 2522, Australia; E-Mails: fjtk886@uowmail.edu.au (F.K.); kwalton@uow.edu.au (K.W.); amcmahon@uow.edu.au (A.M.)

**Keywords:** diet, intake, protein, thiamine, cognition, elderly

## Abstract

The effectiveness of nutritional interventions to prevent and maintain cognitive functioning in older adults has been gaining interest due to global population ageing. A systematic literature review was conducted to obtain and appraise relevant studies on the effects of dietary protein or thiamine on cognitive function in healthy older adults. Studies that reported on the use of nutritional supplementations and/or populations with significant cognitive impairment were excluded. Seventeen eligible studies were included. Evidence supporting an association between higher protein and/or thiamine intakes and better cognitive function is weak. There was no evidence to support the role of specific protein food sources, such as types of meat, on cognitive function. Some cross-sectional and case-control studies reported better cognition in those with higher dietary thiamine intakes, but the data remains inconclusive. Adequate protein and thiamine intake is more likely associated with achieving a good overall nutritional status which affects cognitive function rather than single nutrients. A lack of experimental studies in this area prevents the translation of these dietary messages for optimal cognitive functioning and delaying the decline in cognition with advancing age.

## 1. Introduction

Dementia is a condition in which cognitive and physical functionality gradually deteriorates, resulting in diminished self-care ability. The increasing burden of dementia impacts heavily on already stretched health care services, as well as adversely affecting quality of life and independence of affected older adults and their caregivers. The effectiveness of nutritional interventions to maintain optimal cognitive functioning and prevent cognitive decline in older adults has been gaining interest [1].

In terms of specific nutrients, those most intensively studied for a potential cognitive-enhancing effect include omega-3, antioxidants and B-vitamins [2,3,4]. More recently, intake of combinations of foods consumed within defined dietary patterns, in particular, the Mediterranean diet has been suggested to hold promise [5]. Protein-energy malnutrition is particularly common in the older population and has been found to be related to increased risk of sacropenia and frailty [6]. Nutrition plays an important role in the prevention of sacropenia and frailty which has been suggested to be highly associated with cognitive impairment [7]. A study investigating the nutritional status of hospitalized patients showed high rates of poor nutrition status (malnourished or at risk of malnutrition) in dementia and mild cognitive impairment (MCI) patients in comparison to patients with no cognitive impairment suggesting higher prevalence of malnutrition in older adults with cognitive impairment [8]. A study on young healthy men showed that a high protein diet improved both physical and cognitive function [9] but the effects are still unknown in older populations who may metabolize protein and amino acids less efficiently than younger people [10]. In that study, however, nutritional supplements provided the protein source which limits translation into food-based messages [9].

Thiamine or Vitamin-B1 has been suggested to be associated with neurodegenerative diseases through its role in oxidative and glucose metabolisms [11,12]. Thiamine deficiency is a common condition in older adults especially in hospitalized and institutionalized patients [13] and has also been suggested to be associated with a higher proportion of falls, Alzheimer Disease (AD) and depression [14]. Additionally, post-mortems show decreased activity of two major thiamine dependent enzymes in AD patients compared to age and gender matched cognitively intact controls [15]. In a review of randomized controlled trials involving high-dose thiamine supplementation of >3 mg/day, outcomes on cognitive function were inconsistent [16]. Overall, the evidence was not convincing due to small sample sizes and the use of inappropriate study design such as a cross-over study, which is unsuitable for progressive diseases, such as AD [17]. The aim of the current systematic literature review was to evaluate the available evidence regarding the association between either dietary protein or thiamine and cognitive function in healthy older adults.

## 2. Methods

### 2.1. Identification of Studies

A computerised literature search was conducted using the electronic databases of Cinahl, Medline, Science Direct, Web of Science and Scopus to identify studies that had been published up to mid-May 2014. The primary search terms used in the search strategy included the usage of Booleans (diet* **OR** nutri* **OR** food) **AND** (pattern* **OR** intake* **OR** protein **OR** thiamin*) **AND** (cogniti* **OR** mental **OR** brain **OR** Alzheimer* **OR** dementia) **AND** (elderly **OR** older **OR** age*). The review followed the PRISMA guidelines [18]. No restrictions were applied for type of study, year of publication, before the cut-off date or country.

Inclusion criteria: (i) Older adults (≥60 years), (ii) Human studies, (iii) Relatively healthy subjects (e.g., Absence of cancer, cardiovascular, renal and liver diseases), (iv) Dietary Intake (Not supplements), (v) English language, (vi) Free of significant cognitive impairment.

### 2.2. Data Extraction

Information regarding the study design, demographics of the population, type of cognitive and dietary measurements and results were extracted and tabulated into a summary table. This was conducted based on the evidence analysis manual from the American Dietetic Association (ADA) [19]. The National Health and Medical Research Council (NHMRC) [20] levels of evidence were used to define the type of study, while the quality criteria checklist from the ADA was used to rate the quality of the studies reviewed. No authors were contacted for missing data.

## 3. Results

**Figure 1:**
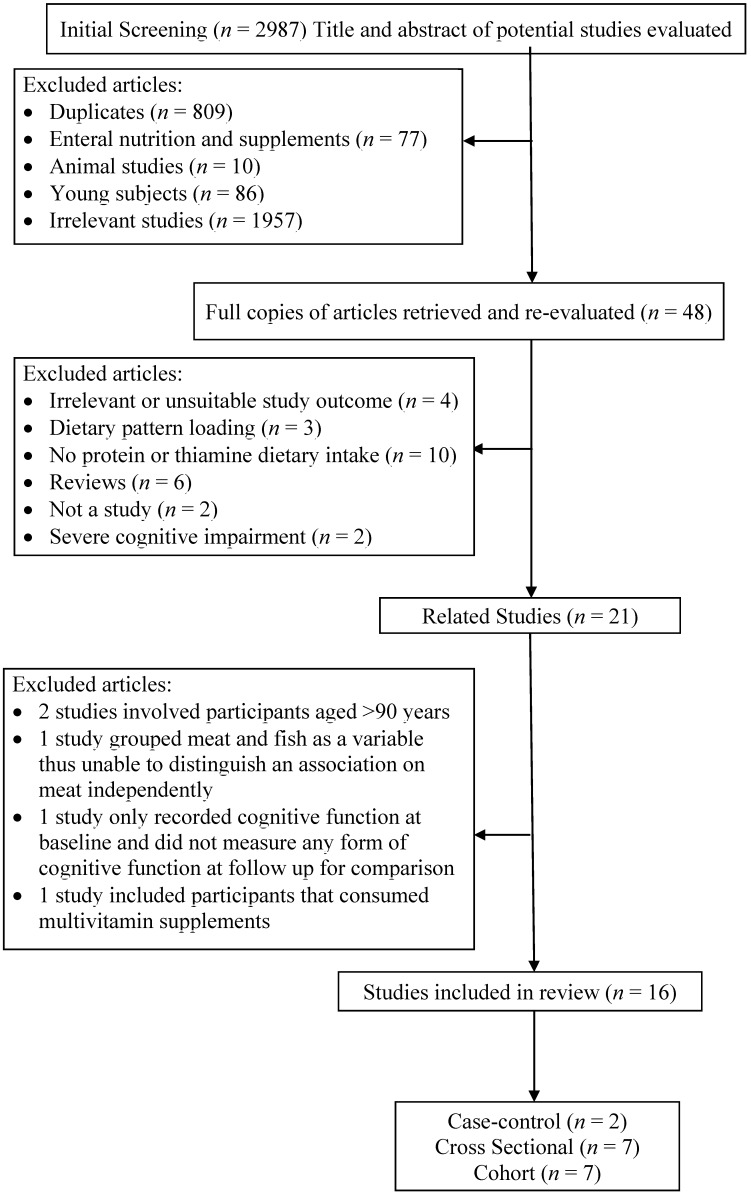
Flowchart of article selection process.

Of the 2987 studies that were identified during the search process, 20 studies met the inclusion criteria. From these, 16 studies were reviewed in full, as shown in Figure 1. Two studies were excluded because they were conducted on people aged ≥90 years and were deemed to be unsuitable because of potential survival bias and lack of generalizability of the results [21,22]. A cross-sectional study was excluded as meat and fish were collated within the same food category [23]. Fish has been suggested to have a protective effect on cognitive decline due to its polyunsaturated fat content [24] One cohort study did not provide follow-up results on cognitive function of the subjects and was therefore excluded [25]. In another cohort study, the reported dietary intakes by participants included supplementation [26]. The eligible studies were all observational in design, including six cohort studies, two case-control studies and seven cross-sectional studies. The most common diagnostic tool used for assessment of cognitive function was the Mini-Mental State Examination (MMSE). Dietary assessment methods employed in the reviewed studies were either one or a combination of Food Frequency Questionnaires (FFQ), food records or 24-hour recalls. The collated papers included in the review provided data for a total of 18,302 study participants.

A detailed appraisal of the included studies summarized according to type of study design is attached as Table 1 for cohort studies, Table 2 for case-control studies and Table 3 for cross-sectional studies, while quality rating outcomes are shown in Table A1.

**Table 1:** Evidence from cohort studies according to year of publication.

Author	Population	Measurement of Cognition (Cutoff Point) and Diet	Protein	Protein Food Source	Thiamine	Adjustments	NHMRC Level of Evidence
La Rue *et al.* 1997 [27], USA	304 community-dwelling healthy individuals, age between 66 and 90 years (6 years cohort).	The Abstraction Scale from Shipley-Hartford Intelligence Test, The Logical Memory and Visual Reproduction subtests from Wechsler Memory Scale and Rey-Osterrieth Test 3-day Food Dietary records	Protein (g/day) Baseline (median): 75 g/day 6 Years Follow Up (median): 72 g/day Rey Osterrieth Recall Test *r* = 0.19 ***p*-value < 0.05** Logical Memory Test *r* = 0.20 ***p*-value < 0.05** Significantly positive association between dietary protein intake and Rey Osterrieth Recall and Logical Memory Test scores.		Thiamine (mg/day) Baseline (median): 1.93 6 years Follow up (median): 2.47 Rey Osterrieth Copy Test Baseline, *r* = −0.07 6 years Follow up, *r* = 0.16 ***p*-value < 0.10** Rey Osterrieth Recall Test Baseline, *r* = 0.08 6 years Follow up, *r* = 0.15 ***p*-value < 0.10** Shipley Hartford Abstraction Test Baseline, *r* = −0.08 6 years Follow up, *r* = 0.29 ***p*-value < 0.01** Significant positive association between dietary thiamine intake and Shipley Hartford Abstraction Test scores.	Body weight	III-2, Neutral
Deschamps *et al.* 2002 [28], France	125 community-dwelling non-demented elderly, age >68 years (5 years cohort) 3-day food diary, Diet history and FFQ	MMSE (cognitive decline: reduction of ≥3 MMSE score over 5 years)	Protein Median intake: 1.33 g/kg/day <1.0 g/kg/day (*n* = 21), OR = 1.00 ≥1.0 g/kg/day (*n* = 104), OR = 1.92 (0.38–9.62) ***p*-value n.s** No significant association between dietary protein intake and cognitive decline.			Age, Gender and Education	III-2, Neutral
McNeill *et al.* 2011 [29], UK	882 subjects living around Edinburg, mean age 70 years. (57 to 60 years cohort)	MHT, MMSE, NART, Verbal Fluency test, Wescler Adult Intelligence Scale–III. Semi-quantitative FFQ			Thiamine (mg/day) Mean Intake from diet: 1.51 ± 0.50 MMSE β = 0.057 ***p*-value n.s** Verbal fluency β = 0.004 ***p*-value n.s** No significant association between dietary thiamine intake and cognitive function or verbal fluency.	Age, gender, IQ at age 11 years, smoking, social class, education, statin use, presence of APOE allele	III-3, Neutral
Barberger-Gateau *et al.* 2007 [24], France	8085 free-living non-demented elderly, age >65 years (4 years cohort)	DSM- IV by neurologist FFQ		Meat All cause dementia 2–3 times/week: Incidence = 1.13 (0.88–1.97), 4–6 times/week: Incidence = 0.85 (0.68–1.01), Daily: Incidence =1.03 (0.80–1.27) ***p*-value n.s** AD 2–3 times/week: Incidence = 0.76 (0.58–0.96) 4–6 times/week: Incidence = 0.53 (0.40–0.66), Daily: Incidence =0.65 (0.46–0.84) ***p*-value n.s** No significant association between meat consumption and dementia/AD.		Age	III-2
Velho *et al.* 2008 [30], Portugal	187 free-living elderly participants with normal cognition, age >65 years (8.5 ± 3.5 months cohort).	MMSE (Improvement: Increase >0 in MMSE score, No Improvement: No increase in MMSE score) 3-day food diary	Protein (g/day) No improvement: 70.9 ± 2.0 Improvement: 73.4 ± 1.8 *t*-test = 1.04 ***p*-value n.s** No significant association between dietary protein intake and improvements in cognitive function.			Age, Total energy	III-2, Neutral
Vercambre *et al.* 2009 [31], France	4809 elderly women from E3N cohort, age between 63 and 68 years (13 years cohort).	DECO scale (<33), Questionnaire for close relative/friend 208 item FFQ, 24 h recalls	Protein (g/day) Mean = 87.70 ± 24.55 Q3-Q1 OR = 0.92 (0.74–1.14) Trend (***p*-value n.s**) No significant association between dietary protein intake and cognitive function.	Beef, pork, lamb (g/day) Mean = 45.45 ± 35.53 Q3-Q1 OR = 0.87 (0.66–1.15) Trend **(*p*-value n.s)** Poultry (g/day) Mean = 16.93 ± 17.89 Q3-Q1 OR = 0.73 (0.58–0.91) Trend **(*p*-value = 0.004)** No significant association for beef, pork and lamb. Significantly higher poultry intake in participants with better cognition.		Age, Education, BMI, Frequency of average physical activity, Average daily energy intake, Smoking, Supplements, Post-menopausal hormones, Depression, Cancer, CHD, Stroke, T2DM, High cholesterol and Hypertension.	III-2, Neutral
Roberts *et al.* 2012 [32], USA	937 cognitively normal participants, age between 70 and 89 (median of 3.7 years cohort)	CDR, Short test of mental status with 9 test assessing 4 domains of memory, executive function , language and visuospatial skills 128 item FFQ	Protein (g/day) All participants (mean): 78 g/day, 18% energy Q1 (20% energy) HR = 0.79 (0.52–1.20) Correlation of trend across quartiles, ***p*-value = 0.03** Significant association between dietary protein intake of 16%–20% of energy intake and reduced risk of MCI or dementia.			Gender, Education, Total daily energy, Non-participation at baseline, Single macronutrient, APOE e4, T2DM, Depression, BMI, Stroke, Marital status, Smoking, Alcohol, Occupation and Frequency of moderate physical activity	III-2, Neutral

Abbrevations: AD, Alzheimer’s Disease; CDR, Clinical Dementia Rating; DECO, Deterioration Cognitive Observée; DSM, Diagnostic and Statistical Manual of Mental Disorders; FFQ, Food Frequency Questionnaire; MHT, Moray House Test; MMSE, Mini Mental State Examination; NART, National Adult Reading Test.

**Table 2:** Evidence from case-control studies according to year of publication.

Author	Population	Measurement of Cognition (Cutoff Point) and Diet	Protein	Protein Food Source	Thiamine	Adjustments	NHMRC Level of Evidence
Burns *et al.* 1989 [33], UK	78 elderly subjects (28 community-living demented, 21 hospitalized demented, 29 control)	MMSE (control: ≥29, case: ≤24) 3-day weighed food record	Protein (g/day) Community living demented: 61 ± 12.0 Control: 44 ± 15.2 ***p*-value < 0.05** Hospitalized demented: 73 ± 8.3 Control: 44 ± 15.2 ***p*-value < 0.05** Significantly higher dietary protein intake in controls than demented participants.			None	III-3, Positive
Nes *et al.* 1998 [34], Norway	32 community-living elderly (16- case, 16-control), age >75 years.	DSM-III 3-day weighed food record	Protein (g/day) Men (Control =75 ± 12, Dementia = 69 ± 14) ***p*-value n.s** Women (Control = 64 ± 14, Dementia = 51 ± 12) ***p*-value ≤ 0.05** Significantly higher dietary protein intake in women controls than women with dementia. No significant difference detected for men.		Thiamine (mg/day) Men (Control =1.0 ± 0.1, Dementia = 1.0 ± 0.1) ***p*-value n.s** Women (Control = 1.0 ± 0.3, Dementia = 0.7 ± 0.2) ***p*-value ≤ 0.05** Significantly higher dietary thiamine intake in women controls than women with dementia. No significant difference detected for men.	None	III-3, Positive

Abbrevations: DSM, Diagnostic and Statistical Manual of Mental Disorders; MMSE, Mini Mental State Examination.

**Table 3:** Evidence from cross-sectional studies according to year of publication.

Author	Population	Measurement of Cognition (Cutoff Point) and Diet	Protein	Protein Food Source	Thiamine	Adjustments	NHMRC Level of Evidence
Ortega *et al.* 1997 [35], Spain	260 free living elderly (108 men and 152 women) aged between 65 and 90 years.	MMSE (unsatisfactory: 0) 7-day weighed food record, FFQ	Protein (g/day) Men (Unsatisfactory MMSE: 81.0 ± 21.3, Satisfactory MMSE: 81.8 ± 18.7) ***p*-value n.s** Women (Unsatisfactory MMSE: 71.8 ± 14.5, Satisfactory MMSE: 73.9 ± 18.8) ***p*-value n.s** No significant association between dietary protein intake and cognitive function.		Thiamine (mg/day) Men (Unsatisfactory MMSE: 1.18 ± 0.25, Satisfactory MMSE: 1.19 ± 0.36) Women (Unsatisfactory MMSE: 0.96 ± 0.26, Satisfactory MMSE: 1.06 ± 0.36) **r = 0.2225, *p*-value < 0.01** Significantly higher dietary thiamine intake in participants with satisfactory scores.	Age, Gender	IV, Neutral
Lee *et al.* 2001 [36]^,^, Korea	449 free-living participants (210 men & 239 women, age >60 years.	MMSE-Korean Version (Poor: ≤19, Inadequate: 20–23, Normal: ≥24) 24-hour recall	Protein (g/day) Men (Normal: 65.1 ± 25.7, Inadequate: 63.9 ± 26.4, Poor: 60.0 ± 25.0) ***p*-value n.s** *r* = 0.078, ***p*-value n.s** Women (Normal: 57.0 ± 24.5, Inadequate: 58.4 ± 29.1, Poor: 42.5 ± 22.3) ***p*-value < 0.05** *r* = 0.181 **(*p*-value < 0.01)** Significant association between higher dietary protein intake and better cognitive function only in women.	Meat (g/day) Men (Normal: 39.2 ± 47.4, Inadequate: 40.9 ± 50.2, Poor: 46.7 ± 47.3) ***p*-value n.s** *r* = −0.004, ***p*-value n.s** Women (Normal: 37.7 ± 52.5, Inadequate: 34.6 ± 57.1, Poor: 20.7 ± 31.1) ***p*-value n.s** *r* = 0.096, ***p*-value n.s** No significant association between meat intake and cognitive function.	Thiamine (mg/day) Men (Normal: 0.95 ± 0.35, Inadequate: 0.91 ± 0.34, Poor: 0.82 ± 0.27), *r* = 0.083, ***p*-value n.s** Women (Normal: 0.91 ± 0.39, Inadequate: 0.90 ± 0.63, Poor: 0.71 ± 0.35) ***p*-value < 0.05**, *r* = 0.125, ***p*-value n.s** Significantly higher intake of dietary thiamine in women normal cognition participants than cognitively impaired participants but not significant in men.	Age	IV, Neutral
Requejo *et al.* 2003 [37], Spain	168 free-living elderly at day centres with normal cognition, age between 65 and 90 years.	MMSE (unsatisfactory: <28, satisfactory: ≥28) 7-day food diary and 5-day weighted food record of lunch		Meat (g/day) Age ≥ 75 years (MMSE < 28: 126.3 ± 66.6, MMSE ≥ 28: 98.9 ± 32.1) Age < 75 years (MMSE < 28: 127.6 ± 60.9, MMSE ≥ 28: 138.5 ± 77.7) ***p*-value n.s** No significant association between higher meat consumption with better cognitive function.	Thiamine Age ≥ 75 years (MMSE < 28: 1.05 ± 0.29, MMSE ≥ 28: 0.96 ± 0.23) Age < 75 years (MMSE < 28: 1.05 ± 0.29, MMSE ≥ 28: 1.12 ± 0.34) ***p*-value < 0.1 almost sig** *r* = 0.2332, ***p*-value < 0.01** Significant association between higher dietary thiamine intake and better cognitive function.	None	IV, Neutral
Rahman *et al.* 2007 [38], USA	1056 community dwelling elderly, mean age = 67.	MSQ (cognitive impairment: <9, normal: ≥9) Verbal FFQ (Yes: Once or twice a week, or most days, or everday. No: Less often than once a week, or never.)		Pork, beef, lamb (g/day) Yes (*n* = 904) No (*n* = 152) OR = 1.11 (0.67, 1.84) ***p*-value n.s** Chicken and turkey Yes (*n* = 916) No (*n* = 140) OR= 0.81 (0.48, 1.36) ***p*-value n.s** No significant association between pork, beef, lamb, chicken or turkey.		Age, Gender, Education and Other dietary factors	IV
Mori *et al.* 2010 [39], Japan	179 community dwelling elderly, aged ≥65 years	SF-36 MCS (High MCS and Low MCS based on standardised score classified by age, 60–69 years = 52.0 and ≥70 years = 51.7 ) Semi-quantitative FFQ	Protein (g/day) (High: 74.5 ± 1 Low: 73.5 ± 1.6) ***p*-value n.s** No significant association between dietary protein intake and cognitive function.	Meat (g/day) (High: 44.0 ± 3.3 Low: 55.3 ± 5.3) ***p*-value n.s** No significant association between meat and cognition.		Age, Gender	IV, Neutral
Aparicio Vizuete *et al.* 2010 [40], Spain	178 institutionalised elderly, age ≥ 65 years.	SMPSQ (0 = No error, >0 = Error) 7-day Weighed Food Records	Protein (g/day) Age < P50 years (No error: 71.01 ± 14.30, Error: 67.63 ± 12.68) Age ≥ P50 years (No error: 70.02 ± 12.89, Error: 67.98 ± 10.67) ***p*-value n.s** *r*^2^ = 0.5899 ***p*-value < 0.001** Significantly higher dietary protein and meat intake in participants with better cognitive functioning.	Meat Age < P50 years (No error: 98.14 ± 41.67, Error: 105.55 ± 40.38) Age ≥ P50 years (No error: 93.05 ± 39.81, Error: 88.90 ± 35.73) **p-value n.s** *r*^2^ = 0.1086 ***p*-value < 0.001** Significantly higher meat intake in participants with poorer cognitive functioning.	Thiamine (mg/day) Age < P50 years (No error: 1.11 ± 0.28, Error: 1.10 ± 0.24) Age ≥ P50 years (No error: 1.12 ± 0.25, Error: 1.09 ± 0.25) ***p*-value n.s** *r*^2^ = 0.3180 ***p*-value < 0.001** Significant association between higher dietary thiamine intake and better cognitive function.	Energy intake and Education level	IV, Neutral
Katsiardanis *et al.* 2013 [41], Greece	557 free-living elderly (m = 237, w = 320), age > 65 years	MMSE (cognitive impairment: <24, normal: ≥24), GDS. FFQ and Semi-quantitative FFQ	Protein (g/day) Men (Cognitive impairment: 82.5 ± 28.84, Normal: 81.0 ± 23.57) ***p*-value n.s** OR = 1.36 (0.92–2.02), ***p*-value n.s** Women (Cognitive impairment: 75.5 ± 24.34, Normal: 74.8 ± 28.59) ***p*-value n.s** OR = 0.88 (0.56–1.37), ***p*-value n.s** No significant association between dietary protein intake and cognitive functioning.	Meat and Meat product Men (Cognitive impairment: 24.0 ± 14.67, Normal: 22.0 ± 10.25) ***p*-value n.s** OR = 1.03 (0.84–1.27), ***p*-value n.s** Women (Cognitive impairment: 18.8 ± 11.47, Normal: 19.8 ± 12.27) ***p*-value n.s** OR = 0.96 (0.81–1.16), ***p*-value n.s** No significant association between meat and meat products with cognitive function.	Thiamine Men OR = 1.05 (0.76–1.44) ***p*-value n.s** Women OR = 1.16 (0.65–1.38) ***p*-value n.s** No significant association between dietary thiamine intake and cognitive function.	Age, Education, Social Activity, Smoking, Metabolic syndrome, Geriatric Depression Scale and MedDiet Score. OR adjusted for core models and energy intake.	IV, Neutral

Abbrevations: DSM, Diagnostic and Statistical Manual of Mental Disorders; GDS, Geriatric Depression Scale; MMSE, Mini Mental State Examination; PMSQ, Pfeiffer’s Mental Status Questionnaire; FFQ, Food Frequency Questionnaire; SF-36 MCS, Short Form (36) Mental Component Score; SPMSQ, Short Portable Mental State Questionnaire.

### 3.1. Dietary Protein Intake

Out of the 11 studies, only six studies showed positive association between dietary protein intake and cognitive function [27,32,33,34,36,40]. Two out of the six studies were associations found in women, the study by Lee *et al.* [36] reported significantly lower protein intake in subjects with poor cognitive function and a positive association between protein intake and Mini Mental State Examination for Koreans (MMSE-K). Nes *et al.* [34] also reported women with dementia to have significantly lower protein intake compared to the control group. A study found significant association between dietary protein intake with specific cognitive components such as verbal and nonverbal learning and memory [27].

### 3.2. Different Types of Protein Food Sources

Eight of the reviewed studies investigated meat protein sources intake as a variable. Of these, only one study found a significant correlation between a higher meat intake and poorer cognitive functioning [40] while the remaining studies found no significant association in either direction. One study found that the incidence of AD was the lowest when meat was consumed 4–6 times/week, compared to when meat was consumed daily or ≤3 times/week [24]. However, there was no significant association between risk for all-cause dementia and consumption of meat [24].

### 3.3. Dietary Thiamine Intake

Based on available mean dietary intake of thiamine from the eligible studies, the mean dietary thiamine intake ranged between 0.7 and 1.51 mg/day. Seven of the nine studies regarding thiamine intake reported a significant association with higher intakes relating to better cognitive function [26,27,34,35,36,37,40]. The study by La Rue *et al.* [27] showed a positive association between dietary thiamine intake and abstract reasoning however there was no significant association between dietary thiamine intake and visuospatial skills or nonverbal learning and memory. In one study, women with poor cognition were shown to have significantly lower thiamine intake than women with normal cognition however there was no significant association between dietary thiamine intake and MMSE-K scores [36]. There are difficulties in interpreting the study outcomes as one of the two studies that reported no significant association was observed to have the highest mean thiamine intake among the other recorded means [29] while the other negative study did not report mean thiamine intake [41].

## 4. Discussion

The evidence regarding the association between dietary protein and cognitive function is weak which is similar to the findings of the previous literature review conducted by Van de Rest *et al.* [42]. Additionally, it is difficult to isolate the effects of protein on cognitive function as protein intake is directly associated with energy intake contributing to a potential effect through associations with nutritional status. Only four studies included adjustments for total energy, three of which showed no significant association after these adjustments. The only study that showed an association suggested that there was a protective effect when protein intake contributed 16%–20% of total energy intake, a level of intake that is considered to be a moderately high [32].

Even at the same protein content, different foods may impact cognition differently because of their specific macronutrient profile (amino acids and fatty acids composition) or micronutrient content (iodine, thiamine, folic acid, vitamin B_12_, *etc*.). As protein is available in various sources of foods, we investigated further to report the evidence available for specific meat sources however the evidences available were very weak, limited and no trends were identified. Although meat is a high protein food, the amounts of saturated fat present is highly dependent on the type of meat and the cut. A majority of the reviewed studies in relation to meat products found no significant association. One cross-sectional study reported higher meat consumption to be associated with poorer cognitive functioning [40]. However, in that study the association between meat intake and errors identified in Short Portable Mental State Questionnaire (SPMSQ) differed according to age of participants. A beneficial association was found between a higher meat intake and better performance in those aged below the median, while in those whose age was above the median, the association was in the opposite direction. [40]. Curiously, the authors did not perform analyses, which included age as a continuous variable, which is a limitation of the study [40].

Literature related to thiamine intake presented inconsistent findings. Those studies that reported an association between higher intake and better cognition were mainly cross-sectional. Only two cohort studies were identified and one showed a positive association [27] while the other study showed no association between dietary thiamine intake and cognitive function [29]. The overall health status of the study participants also needs to be considered as lifestyle factors such as physical activity, smoking, alcohol consumption and even anthropometric characteristics are well known potential confounding factors that are related to both dietary intake and cognitive function.

Our findings were somewhat surprising. Thiamine is an essential nutrient involved in brain metabolic and cellular functions, including carbohydrate metabolism and neurotransmitter production, notably acetylcholine and gamma-amino butyric acid (GABA). Reduced synthesis of acetylcholine leads to cognitive disorders such as delirium [43] while thiamine deficiency in older adults is associated with depression and Alzheimer’s Disease [14]. Our present review excluded studies of older people with marked cognitive dysfunction. Thus, the role of thiamine may only be evident in patients with senile dementia. Indeed, Alzheimer’s patients tend to have lower plasma thiamine concentrations and higher rates of thiamine deficiency compared to patients without SDAT [44,45]. It has been suggested from animal studies that thiamine deficiency damages neurons and results in tissue loss in the brain [46]. It has been hypothesised that therapeutic doses of thiamin intake may be beneficial in the treatment of neurodegenerative diseases [16], however our review does not support that view for healthy older adults.

The limited number of studies and a lack of randomized controlled trials indicate that there is insufficient evidence to draw conclusions regarding the role of either protein or thiamine on cognitive function in older adults. Observational data, apart from sufficiently long duration prospective cohort studies, of which there were only seven, is unable to provide information on temporality, in order to determine whether the intake or lack of intake of a nutrient caused cognitive decline or if the progressive cognitive decline caused the reduced intake. The appropriate type of study design is often questioned as cognitive function is also known to decrease with age and dementia is a progressive disease. For instance, nutritional supplementation is often used instead of food sources in experimental studies due to their convenience, cost effectiveness and higher levels of control during interventions. However, provision of nutrients within a food matrix is likely to be more effective than single nutrients alone as has been shown for the relationship between vitamin E and prostate cancer. In a large prospective study, γ-tocopherol, a form of vitamin E obtained through diet had a protective effect against prostate cancerin comparison to α-tocopherol, the more commonly available form of supplemental vitamin E that showed no association to prostate cancer risks [47]. Another study showed that supplemental vitamin E had no effect on reducing risk of prostate cancer in healthy men and was suggested to have adverse effects at high doses [48].

Comparison of the study outcomes is limited by the variety of cognitive tests used. The MMSE was the most preferred and common form of cognitive testing, however reference cut-off points differ between studies and population groups. Most of the studies included cognitive tests that had been validated for their study population except for two studies which had no information provided regarding this issue [27,28]. Additionally, generalizability of the findings is hampered by the diversity of study populations and their different dietary patterns.

## 5. Conclusions

There is insufficient evidence to support an association between dietary intake of either protein and/or thiamine and cognitive functioning in healthy older people. A lack of experimental studies prevents the translation of information into dietary messages for optimal cognitive functioning. Undoubtedly, there is a need for further well designed cohort and experimental studies in this area.

## Appendix

**Table A1:** Quality rating process.

*Study (1st Author, Year)*	Nes *et al*. 1988	Burns *et al*. 1989	Ortega *et al*. 1997	La Rue *et al.* 1997	Lee *et al.* 2001	Deschamps *et al.* 2002	Requejo *et al.* 2003	Barberger *et al.* 2007	Rahman *et al.* 2007	Shatenstein *et al.* 2007	Velho *et al.* 2008	Vercambre *et al.* 2009	Aparicio Vizuete *et al.* 2010	Mori *et al.* 2010	McNeill *et al.* 2011	Roberts *et al.* 2012	Katsiardanis *et al.* 2013
*Reference Number*	[34]	[33]	[35]	[27]	[36]	[28]	[37]	[24]	[38]	[26]	[30]	[31]	[40]	[39]	[29]	[32]	[41]
**VALIDITY QUESTIONS—PRIMARY STUDIES**																	
*1. Was the research question clearly stated?*	Y	Y	Y	Y	Y	Y	Y	Y	Y	Y	Y	Y	Y	Y	Y	Y	Y
*2. Was the selection of study subjects/patients free from bias?*	Y	Y	N	Y	Y	Y	Y	Y	Y	Y	N	Y	Y	N	Y	Y	Y
*3. Were study groups comparable?*	Y	Y	NA	NA	NA	NA	NA	N	NA	Y	NA	NA	NA	NA	NA	NA	NA
*4. Were intervention/therapeutic regimens/exposure factor or procedure and any comparison(s) described in detail? Were intervening factors described?*	Y	Y	Y	Y	Y	N	Y	N	N	Y	Y	Y	Y	Y	Y	Y	Y
*5. Were outcomes clearly defined and the measurements valid and reliable?*	Y	Y	Y	Y	Y	Y	Y	Y	Y	Y	Y	Y	Y	Y	Y	Y	Y
*6. Was method of handling withdrawals described?*	Y	N	N	N	N	Y	N	N	N	Y	Y	Y	N	N	N	Y	Y
*7. Was blinding used to prevent introduction of bias?*	N	Y	N	N	N	N	N	N	N	N	N	N	N	N	N	N	N
*8. Was the statistical analysis appropriate for the study design and type of outcome indicators?*	N	N	Y	Y	Y	Y	N	Y	Y	N	Y	Y	Y	Y	Y	Y	Y
*9. Were conclusions supported by results with biases and limitations taken into consideration?*	Y	N	Y	Y	Y	Y	N	Y	Y	Y	Y	Y	Y	Y	Y	Y	Y
*10. Is bias due to study’s funding or sponsorship unlikely?*	Y	Y	Y	Y	Y	Y	Y	Y	Y	Y	Y	Y	Y	Y	Y	Y	Y
**OVERALL QUALITY—PRIMARY STUDIES**																	
Negative/Neutral/Positive **(N/0/P)**	P	P	0	0	0	0	0	0	0	P	0	0	0	0	0	0	0
**If most (six or more) of the answers to the above validity questions are “No,” the report should be designated negative**																	
**If the answers to validity criteria questions 2, 3, 6, and 7 do not indicate that the study is exceptionally strong, the report should be designated neutral**																	
**If most of the answers to the above validity questions are “Yes” (including criteria 2, 3, 6, 7 and at least one additional “Yes”), the report should be designated positive**																	
**Sum**																	
Yes (Y)	7	7	6	7	7	7	5	6	8	8	7	8	7	6	7	8	8
No (N)	3	3	3	2	2	2	4	5	3	2	2	1	2	3	2	1	1
Not Applicable (NA)	0	0	1	1	1	1	1	0	1	0	1	1	1	1	1	1	1
